# Quality of Care for Patients With Traction in Shahid Beheshti Hospital in 2012

**DOI:** 10.5812/atr.9127

**Published:** 2013-08-01

**Authors:** Mohsen Adib Hajbaghery, Tayebeh Moradi

**Affiliations:** 1 Trauma Nursing Research Center, Kashan University of Medical Sciences, Kashan, IR Iran; 2 Student Research Committee, Faculty of Nursing and Midwifery, Kashan University of Medical Sciences, Kashan, IR Iran

**Keywords:** Quality of Health Care, Patients, Skeletal Traction, Skin Traction

## Abstract

**Background:**

With increasing incidence of traumatic fractures, the use of orthopedic intervention such as traction has increased. Inappropriate traction care may cause substantial morbidity and delay the patient rehabilitation.

**Objectives:**

This study was conducted to evaluate the quality of care for patients with traction in the orthopedic unit of Kashan's Shahid Beheshti Hospital, Kashan, Iran.

**Patients and Methods:**

This observational study was conducted on 100 patients with traumatic fractures of hip and femur bones who were admitted to Kashan Shahid-Beheshti Hospital during the first 6 months of 2012, and for whom skeletal or skin traction was performed. Data were collected using a checklist including questions about the personal characteristics and 23 items related to care for patients with tractions. These items were in three domains including caring while establishing traction, recording care and patient’s education. Descriptive statistics were calculated and data were analyzed using the independent sample t-test and Pearson correlation coefficient.

**Results:**

The mean age of patients was 51.16 ± 23.28 years and 66% of them were male. In total, 47% of the patients were treated by skin traction and 53% by skeletal traction. The overall mean score of quality of care was 10.20 ± 2.64. Quality of establishing traction was good in 55% of patients, but the quality of care was poor in the domains of recording care (88%) and patient education (96%). Total mean of quality of care was significantly different between male and female patients (P < 0.02).

**Conclusions:**

The quality of care of patients with traction was not optimal. Therefore it is necessary to improve measures in this area.

## 1. Background

Modern industrialized life and increasing incidents of road accidents and other incidents have led to an increased incidence of fractures (1). Each year, more than 340,000 cases of hip fractures occur in America and 1.6 million cases world wide, out of which 13% to 37% lose their lives (2). Ninety percent of these fractures occur in individuals older than 50 years old. In younger patients, fractures are usually the result of high-energy physical traumas such as motor vehicle accidents and usually occur in the absence of any underlying disease (2, 3). Morbidity and mortality of these fractures are high. Following hip fractures, 50% of patients are unable to walk without aid, 25% require long-term care, and 20% die during the first year (4, 5). Given that Iran stands among Asian countries in which bone density in individuals has been reported to be lower than average, and due to the increasing number of elderly, the rate of fractures will also increase in this country (6, 7). Traction is a common method used for the treatment of patients with hip fractures and is applied two ways: skin traction or skeletal traction. Tractions are usually used before surgery to reduce pain and facilitate the process of surgery (8). Severe complications such as damage to the neurovascular structure, physical damage, ligamental damage, and pin loosing and infection in pin tract may occur following skeletal traction (9). Also in skin traction, pressure exerted on the skin can cause skin damage and there is a risk of ischemia (10). Other possible adverse effects of skin traction are damages to the skin by mechanical shearing, ischemia to the limb from tight bandages or allergy to adhesive strapping (9). In one study, it was found that pin tract infection rate was 20% and the rate of pin loosing was 15% (9). Rates for pin tract infections were reported to be 11.2% to 63%, by other studies (11-14). Because of the prevalence of femur and hip fractures, several studies have focused on these fractures (3, 4), and its hospital costs (6). One study also focused on the experiences of undergraduate nursing students in orthopedic units (15). Another study investigated the functional outcomes after hip fractures (2). Few studies also focused on patients under traction and reviewed or investigated the benefits, indications, techniques, and complications of skeletal or skin tractions (8-10) and pin tract infection rates (13, 14). However, only one study was found that focused on the nurses’ knowledge and practice concerning care of patient with skin traction in Iraq and reported a poor performance (16). Although the lack of care for patients with traction can cause substantial morbidity and can delay rehabilitation of the individual patient (17), the above review indicated that the quality of care of patients with traction has not been investigated enough and has been largely ignored. Thus, an important gap exists in this area. Given the increased prevalence of fractures and consequently increased use of traction, and additional costs of complications resulting from improper care, and considering the lack of studies in the area of quality of care of orthopedic patients and specially patients with tractions, health care staff in orthopedic units are responsible to pay attention to care for these patients.

## 2. Objectives

Due to the lack of studies on quality of care of patients with traction, this study was conducted to evaluate the quality of care of patients with traction in the orthopedic unit of Shahid Beheshti hospital, Kashan, Iran.

## 3. Patients and Methods

This observational prospective study was conducted on patients with traumatic fractures of hip and femur bone admitted to Kashan Shahid-Beheshti Hospital during the first 6 months of 2012, and skeletal or skin traction was performed on them. All patients with the inclusion criteria (as described in the previous sentence) that were fully conscious and consented to participate in the study were entered in the study. Data were collected using a checklist consisted of two parts. The first part consisted of 4 questions regarding personal characteristics including age, gender, duration of hospitalization and the type of traction. The second part included 23 items, with a ‘yes = 1’ or ‘no = 0’ format, related to the quality of care for patients with traction. These questions were in three domains including caring while establishing traction (13 Items), recording the care delivered (6 Items) and patient education (4 items). Data regarding personal characteristics were collected using the patients’ filed records. The quality of establishing traction, quality of recording the care delivered, and the quality of patient education were examined through direct observation, reviewing the nursing reports in the patients' files, and by interviewing the patients, respectively. Scores for quality of establishing traction ranged from zero to 13. Score from zero to three were classified as poor, 4 to 8 as average, and 9 to 13 were classified as good, respectively. In the domain of ‘recording the care delivered’, scores from zero to one were considered as poor, 2 to 4 as moderate, and 5 to 6 as good. In the domain of patient education, scores from zero to one were considered as poor quality of care; 2 to 3 as moderate, and 4 as good. Overall quality of nursing care of patients with traction was calculated by adding all scores of the three areas. Scores from zero to seven were classified as poor quality of care; 8 to 16 as moderate and 17 to 23 as good. The checklist was developed through an extensive review of related contents in nursing textbooks and literature related to care of orthopedic patients (17-20). Content validity of the checklist was confirmed by a number of nursing faculty members in Kashan University of Medical Sciences. Reliability of the checklist was studied using a pilot study on 10 patients and reliability coefficient was calculated using Kuder–Richardson Formula 20 (KR - 20) that was 0.73 for the whole instrument. The second author collected all data, for the 6 months duration of the study through the participant observation method. During this period, the second researcher was present in the ward as a nurse instructor and could observe the nurses when they were caring for their patients. All the information was collected during the morning and evening shifts. The checklist was filled on the day of the start of traction (for patients with skin or skeletal traction who had a hip or femur fracture due to trauma or accident) when the patients were fully conscious and then rechecked the day after to double check the information, due to the time constraint and workload or because the patient’s condition may have postponed some of the necessary data collection such as patient’s education. Permission for the study was obtained from the authorities in the nursing and midwifery faculty of Kashan University of Medical Sciences, the authorities of the hospital and the ethics committee of the university. The purpose of the study was explained to all patients. They were all assured about the confidentiality of their personal information and that their responses do not affect the care they receive. All of the patients signed an informed consent form before participation in this study. All the observed nurses also signed the informed consent at the end of the study. The SPSS 11.5 software was employed to analyze the data. Descriptive statistics were calculated and independent sample t-test was used to examine the difference between subgroups. Pearson correlation coefficient was used to determine the relationships between variables. A P value less than 0.05 was considered as significant for all tests.

## 4. Results

In this study, 100 patients were evaluated and of these 66 (66%) were male and 34 (34%) were female. The mean age of patients was 51.16 ± 23.28 years. In total, 47 patients had skin traction and 53 had skeletal traction. Average duration of hospitalization of the patients was 2.81 ± 1.9 days. The mean score of overall quality of care for patients with traction was 10.20 ± 2.64 (Table 1). 

**Table 1.** Mean Scores and Standard Deviation for the Three Domains of Quality of Care for Patients With Traction

Domains	Score, Mean ± SD	The Highest Possible Score
**Establishing traction**	8.76 ± 2.97	13
**Recording the care delivered**	0.9 ± 0.7	6
**Patient education**	0.54 ± 0.64	4
**Overall quality of care**	10.20 ± 2.64	23

**Figure 1.**
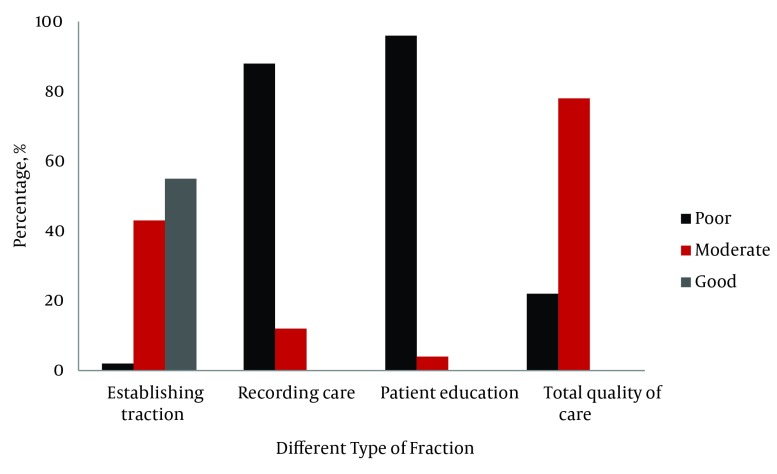
Percentage of the Three Domains of Quality of Care for Patients With Traction

Quality of establishing traction was good for the majority of patients (55%), but the quality of care was mostly poor in the domains of recording the care delivered (88%) and providing patient education (96%) (Figure 1). 

Though the overall mean of quality of care was significantly different between male and female patients (P < 0.02), such a difference was only related to the domain of ‘establishing traction’ (P < 0.007). Also, statistically significant differences were observed between the overall quality of care scores of patients with skeletal and skin traction. However, such difference was not significant in the domain of patient education (Table 2). Weak but significant inverse correlations were observed between the scores of ‘establishment of traction’ and ‘total quality of care’ with the patients’ age. However, significant direct correlations were observed between the scores of ‘establishment of traction’, ‘patient education’ and ‘total score’ for quality of care with the duration of hospitalization (Table 3). 

**Table 2.** Comparison of Quality of Care Scores of Patients With Traction in Terms of Gender and Type of Traction

Domains	Gender			Type of Traction		
	Female, Mean ± SD	Male, Mean ± SD	P value	Skeletal Traction	Skin Traction	P value
**Establishing traction**	7.91 ± 2.53	9.20 ± 2.04	0.007	9.23 ± 2.17	8.23 ± 2.34	0.03
**Recording the care delivered**	0.85 ± 0.61	0.92 ± 0.75	0.63	1 ± 0.73	0.79 ± 0.65	0.09
**Patient education**	0.59 ± 0.74	0.52 ± 0.58	0.59	0.64 ± 0.68	0.43 ± 0.58	0.13
**Overall quality of care**	9.35 ± 2.61	10.64 ± 2.57	0.02	10.87 ± 2.58	9.45 ± 2.54	0.007

**Table 3:** Correlation Between Age and Duration of Hospitalization With the Score of Various Domains of Care

	Establishing Traction	Recording the Care Delivered	Patient Education	Total Quality of Care
Age				
r	- 0.29	0.16	> 0.050	- 0.29
P value	0.003	> 0.05	0.35	0.003
**Duration of Hospitalization**				
r	0.36	0.08	0.35	0.42
P value	0.002	> 0.05	0.003	0.001

## 5. Discussion

In this research, the quality of care for patients with traumatic fractures of hip and femur bone was studied. Results indicated an inadequate quality of care for patients with traction. The overall quality of care for patients with traction was moderate. The study by Al Barwari has reported the poor performance of nurses in Baghdad and Erbil hospitals and that more than 90% of nurses have performed poorly in the nursing care of patients with skin traction (16). In another study, it was found that care provided by orthopedic nursing students is the result of a care relationship that emerges from their sensitivity toward patients and their own knowledge, skills and attitudes (21). Also, it has been reported that the quality nursing and safety of patient care are significantly influenced by the number of nurses in charge in each unit, their workload and the nurse-patient proportions (15). Hallin and Danielson introduce low nurse-patient relationship as an indicator for low quality nursing care (22). Al-Aboudy demonstrated that nurses should increase their knowledge and performance in the field of management of patients in orthopedic departments (16). In this study, the overall mean of quality of care was higher for male than female patients. Also when we consider the three domains of care, such a difference was only related to the domain of ‘establishing traction’. However, it seems that nurses tend to perform better for male patients. This finding should be interpreted cautiously and needs more investigations to be confirmed or rejected, however it may represent a gender difference in nursing care and overall health care as a historical aftermath for paternalism that needs to be overcome (23). Our results showed that the overall mean of quality of care was higher for patients with skeletal traction. This was mostly related to the domain of establishing traction. It seems that care providers, tent to do better for patients with skeletal traction. Perhaps the type of traction is connected with the interpretation of the severity of damage and the demand for care. Thus patients with skin traction may be at a greater risk for traction complications. The results of this study showed that the quality of patient education was poor. Other studies have also reported that the quality of patient education is poor in Iran (24, 25). Although the doctors are not innocent in this regard, such poor conditions may also be attributed to factors such as lack of time for nurses, high number of patients and low number of nurses in charge (26-28). In clinical settings, patient education is an important part of regular and evidence-based nursing care, since high quality patient education promotes active patient participation in healthcare decision-making and helps to improve patients’ and caregivers’ psychological well-being (29). The results of the present study showed a poor performance of nurses in recording the care delivered to patients in traction. Ghazanfary et al., Rozitalab et al. and Cheevakasemsook et al. have also reported that quality of recording the detail of nursing care is poor (30-32). Perhaps, this condition may be due to factors such as lack of time, high workload, and giving priority to direct nursing care as Ghazanfary et al. has reported (31). Perhaps, establishing electronic nursing information systems would not only promote nursing documentation and data entry, but also help nurses to organize their work, manage care plans, track diagnoses and outcomes, and support decision making (33). It has also been shown that nurses need strong managerial support in order to keep a high quality documentation of nursing care (34). Darmer has also showed that spending course of nursing documentation may promote knowledge of nurses about the principles of documentation (35). In the present study, a direct correlation was observed between the quality of care and the duration of hospitalization of patients with traction. It seems that the overall quality of care for patients increases with increased hospital stay. This may be a sign of the time pressure and high workload in nursing. So, nurses postponed some of the care requirements until they find free time.

This study showed that the quality of care for patients with traction is not optimal. Therefore it is necessary to improve measures in this area. By improving patient education through providing enough personnel and nominating one or two nurses for educational purposes, and also through explicating responsibility for the care team members in patient education and training classes, it would be possible to improve performance of nurses in educating patients. Moreover, by holding forums and workshops about nursing care documentation, it would be possible to increase the nurses’ knowledge and priority in this regard. Also, the hospital authorities should provide the standard and evidence based protocols for caring for patients with tractions. Establishment of some re-training programs and strengthening supervisions may also be effective. However, research on the quality of care of patients with traction is very limited and more research is needed to be done in this area.

## Appendix

The checklist designed for assessing the quality of care of patient with traction had been come in the following text (Table 4).

Patient’s age: …………….. year

Patient’s gender: male female

Type of traction: skin traction: skeletal traction

Length of hospitalization: ……………….. days

**Table 4.** Questionnaire Form

Item No.	Items	Yes	No
**1**	Traction and weights are applied in the opposite directions.		
**2**	The patient is in the center of the bed when traction and the affected limb are in good body alignment when traction is applied.		
**3**	The traction ropes are intact and unobstructed (Knots in the rope do not touch the Pulley)		
**4**	Traction installed using intact and free pulleys and the traction rope is fully extended and can move freely on the pulley.		
**5**	The patient’s body weight and bed position supply the needed counter-traction.		
**6**	No factors prevent traction.		
**7**	Weights are not removed unless intermittent traction is prescribed.		
**8**	Weights are hanging freely and do not rest on the bed or floor.		
**9**	The distal portion of the limb with traction is not resting on the foot of the bed.		
**10**	The amount of weight applied must not exceed the tolerance of limb (No more than 2 to 3.5 kg for skin traction, 4.5 to 9 kg for pelvic traction. 7 to 12 kg for skeletal traction).		
**11**	An overhead trapeze is used which is easy to reach for the patient to encourage movement.		
**12**	The bed’s linen is not wrinkled.		
**13**	The ends of the pins are covered with corks or tape to prevent injury to the patient or caregivers. (Skin traction elastic bandages are not very loose or too tight).		
**14**	The neurovascular condition of the limb was assessed and recorded in the nursing notes.		
**15**	In skin tractions: skin condition and its reaction to the traction tape was frequently assessed and recorded in the nursing notes (to ensure that shearing forces are avoided).		
**16**	The patient’s fluid intake and output was frequently checked and recorded.		
**17**	The patient’s defecation and possibility of constipation was frequently checked and recorded in the nursing notes		
**18**	The patient’s lung sounds were frequently checked and recorded in the nursing notes		
**19**	The patient’s education was recorded in the nursing notes		
**20**	The mechanism of traction and the point, at which traction must continue to be effective, was taught to the patient. (asking the patient)		
**21**	The patient was educated about the importance and the appropriate methods for active movements of unaffected limbs. (asking the patient)		
**22**	The patient was educated about the importance and the methods for isometric movements in the affected limb (asking the patient)		
**23**	The patient was educated about the importance of using enough fluids and a high-fiber diet (asking the patient)		
